# Drugs to Treat Alcohol Dependence-A Perspective

**DOI:** 10.15436/2471-061X-16-021

**Published:** 2016-06-06

**Authors:** Joydip Das

**Affiliations:** Department of Pharmacological and Pharmaceutical Sciences, College of Pharmacy, University of Houston, Houston, TX, US

## Abstract

Despite increased awareness, prevention campaigns, and tighter laws and regulations, alcohol consumption caused an average of 88,000 deaths per year and a burden of $249 billion to the U.S economy in 2010. Only three FDA-approved drugs, disulfiram, naltrexone and acamprosate are available for the treatment of alcohol use disorder. These drugs are only effective modestly and patient compliance is a serious issue because of several adverse side effects, necessitating the developments of newer drugs. The current research drug development efforts remains in the identification new alcohol targets and elucidating the molecular mechanism of its action is needed for effective intervention. In addition, current treatment could be improved by testing the existing medications for comorbid conditions using the patient’s genetic background information.

## Introduction

Excessive alcohol consumption is widely recognized for the heavy toll it exacts on the individual, although there is a significant body of research suggesting some health benefits from moderate consumption of alcohol. Health problems related to excessive drinking is termed as Alcohol Use Disorder (AUD). AUD is defined as alcohol abuse and alcohol dependence. Data provided at the National Institute on Alcohol Abuse and Alcoholism (NIAAA) homepage reveal that approximately 7.2% or 17 million adults in the United States aged 18 and older had an AUD in 2012. This includes 11.2 million men and 5.7 million women, and an estimated 855,000 adolescents aged between 12 and 17. Yet, despite increased awareness, countless prevention campaigns, and tighter laws and regulations, an October 2015 Centers for Disease Control and Prevention study revealed that excessive alcohol consumption is responsible for an average of 88,000 deaths per year and a cost of $249 billion to the U.S. economy in 2010^[1]^. Excessive drinking primarily affects two major organs-the brain and the liver. While impairment of cognitive behavior and addiction problems are mediated through the brain, alcohol is metabolized in the liver. So far, only three drugs, disulfiram, naltrexone and acamprosate have got FDA approval for the treatment of alcohol addiction problems. The following is the brief description of these drugs, their mechanism of actions, metabolism and side effects.

### Disulfiram

Disulfiram (Antabuse^®^) is the first drug approved by FDA in 1951 for treating alcohol dependence.

In its metabolism, alcohol or ethanol is first converted to acetaldehyde by alcohol dehydrogenase and subsequently, acetaldehyde is converted to acetic acid by aldehyde dehydrogenase. Disulfiram interferes with the hepatic oxidation of acetaldehyde to acetic acid by inhibiting aldehyde dehydrogenase. It competes with Nicotinamide Adenine Dinucleotide (NAD) for binding on aldehyde dehydrogenase^[2]^. While disulfiram does not alter the rate of elimination of ethanol, it can increase the serum acetaldehyde concentrations five to ten times higher after alcohol consumption. This accumulation of acetaldehyde causes unpleasant reaction in patients. This also can generate a feeling of throbbing headache, dyspnea, nausea, copious vomiting, diaphoresis, thirst, chest pain, palpitation, tachycardia, hypotension, blurred vision, vertigo, anxiety, syncope, confusion, etc. Further, more severe reactions including respiratory depression, arrhythmias, and cardiovascular collapse can also occur.

Disulfiram is administered orally. For adults, starting with 500 mg every morning for 1 – 2 weeks, then reducing to 250 mg daily is the preferred dosage. Dosages ranging from 125 – 500 mg once daily, not exceeding 500 mg/day, is recommended for maintenance. The duration of the treatment depends on the patient’s ability to abstain from ingesting ethanol, ranging from months to year. Slow hepatic metabolism converts disulfiram to diethyl dithiocarbamate, diethylamine, and carbon disulfide. One-fifth of a dose may remain in the body for a week or longer, showing the risk of drug accumulation. Unpleasant effect of disulfiram-alcohol reaction can still persist on drinking even after cessation of the therapy after two weeks.

There have been instances of deaths following the administration of even lower dosages of disulfiram and the ingestion of a single alcoholic drink. Tolerance to the drug has not been a big issue. Disulfiram shows drug-drug interactions. Although disulfiram is relatively nontoxic, it interacts with many drugs that are hepatically metabolized by inhibiting hepatic enzymes such as, cytochrome P450 isoforms CYP2E1 and CYP1A2. This increases serum levels of the drugs and corresponding adverse effects. It may also cause peripheral neuropathy. All these have contributed to the poor compliance of the drug. However, disulfiram can be effective among patients who are highly motivated and patients who use it episodically for high-risk situations.

### Naltrexone

FDA approved naltrexone in 1994 for treating alcohol addiction.

Naltrexone is a morphine derivative that acts as a competitive antagonist at opioid receptors μ, δ and ƙ. Naltrexone can either displace opioid agonists from binding at these receptors or prevent opioid binding. Blockade of opioid receptors by naltrexone is a competitive phenomenon and results in elimination of the euphoric effect of opioid. Recent structural studies of the antagonist bound opioid receptors have provided better understanding of the mechanism of action of the opioid antagonists^[3–5]^. At usual opioid concentrations, naltrexone’s greater affinity for the receptor prevents the binding of the opioid agonist to the receptor. However, when opioid concentrations are extremely high, the opioid can displace naltrexone, and respiratory depression and/or death is possible.

The mechanism of action by which naltrexone prevents alcohol dependence is not understood very clearly. Naltrexone is believed to interfere with the interactions between dopamine and the endogenous opioid neuropeptide systems. Alcohol stimulates release of dopamine from cells originating in the brain’s Ventral Tegmental Area (VTA) region, which is a component of a neuronal circuit called the mesolimbic dopamine system and is associated with behavioral motivation and reward. Following exposure to alcohol, dopamine released into the nucleus accumbens (NAc) and prefrontal cortex reinforces drinking behaviors^[6,7]^. Whereas alcohol does not appear to selectively bind dopamine receptors, its effects on dopamine release are likely mediated through interactions with several neurotransmitter systems, as well as through interactions with the endogenous opioid system, such as endorphins and enkephalins. These endogenous opioids are involved in the expression of alcohol’s reinforcing effects and may promote drug-seeking behaviors. An opioid reward system mediated by μ and δ opioid receptors and an opposing aversions system mediated by ƙ opioid receptors must be in balance to maintain a neutral state in regards to the development of addiction. Naltrexone functions as a competitive antagonist at opioid receptors and inhibits the effects of endogenous opioids and decreases the positive or reward pathways associated with the imbalance caused by alcohol^[8]^.

Naltrexone is administered orally or intramuscularly. Naltrexone is metabolized to 6-β-naltrexol, which also has antagonistic activity but is less potent than the former. Significantly less 6-β-naltrexol is generated following Intramuscular (IM) administration of naltrexone compared to administration of oral naltrexone due to a reduction in first-pass hepatic metabolism. Two other minor metabolites are 2-hydroxy-3-methoxy-6-β-naltrexol and 2-hydroxy-3-methyl-naltrexone. The side effects of naltrexone include development of withdrawal symptoms, nausea, dysphoria and fatigue. Naltrexone also can impair thinking and induce anxiety. A daily dose of 50 mg once with food for 12 weeks has been shown to be effective for adults. Some patients may require naltrexone doses of 100 mg/day. Initially, patients may require 3 – 6 months of naltrexone treatment and some may benefit from up to 1 year of treatment. Starting with a dose of 12.5 – 25 mg once daily and gradually titrating, splitting or adjusting the administration times are recommended to decrease gastrointestinal side effects

An extended release version of naltrexone, vivitrol is administered once a month by injection. FDA approved this drug in 2006 for treating alcoholism, and may offer benefits regarding compliance. For adults, a 380 mg Intramuscular (IM) dose every 4 weeks is effective. Vivitrol is indicated in patients who are able to abstain from alcohol in an outpatient setting prior to treatment initiation

Recently European Medicines Agency (EMA) approved nalmefene, an opioid receptor antagonist for as-needed treatment to reduce heavy drinking in alcohol-dependent patients^[9]^. Structurally, nalmefene is very similar to naltrexone except that the keto group attached to the cyclohexane ring is replaced by a C=C. This chemical modification, however, changes the pharmacokinetics of this drug. Nalmefene shows longer half-life, greater oral bioavailability and has no dose-dependent liver toxicity as compared to naltrexone. A daily dose of 5 – 40 mg of nalmefene has been tested to be effective^[10]^.

### Acamprosate

Acamprosate (Campral^®^) received FDA approval in 2004, although it was approved in France, much earlier, in 1989. Acamprosate reduces alcohol intake in alcohol-dependent animals in a dose-dependent manner^[11,12]^.

The mechanism of action of acamprosate in maintenance of alcohol abstinence is not completely understood. Because of structural similarities with γ-amino butyric acid (GABA), it was hypothesized that acamprosate may act as a GABA-mimic or otherwise modulate GABA-ergic transmission^[13]^. Although it was shown that it may bind to the GABA_A_ and GABA_B_ receptors, there is little in the in vivo profile of acamprosate to suggest similarities with drugs known to enhance GABA-ergic transmission. It is now generally accepted that acamprosate’s mechanism of action is likely to involve modulation of glutamatergic function through partial agonist activity on the NMDA receptor complex via actions at its polyamine site. More recently, it was found that acamprosate blocked neurotoxicity induced by a mGluR agonist with affinity for mGluR1 and mGluR5 receptors^[14]^.

For the mechanism of action on alcohol dependence, it is believed to normalize balance between excitatory and inhibitory pathways in the brain that become adapted to chronic drinking. For example, chronic alcohol consumption may upregulate the excitatory N-methyl-D-aspartate (NMDA) glutamatergic system to overcome the sedative effects of the potentiated inhibitory GABAergic system, allowing the Central Nervous System (CNS) to function more normally in a depressed state^[15]^. This change in the neurotransmitter system also alleviates the withdrawal symptoms such as anxiety, hyper-excitability and insomnia.

Acamprosate is administered orally. The suggested dose for adults is 666 mg (two 333-mg delayed-release tablets) 3 times daily. Alternative dosage regimens such as, doses of 1332 mg/day (666 mg in the morning, 333 mg at mid-day, and 333 mg in the evening) were administered for patients having body weight less or equal to 60 kg, and doses of 1998 mg/day (666 mg, 3 times daily) were administered for patients having body weight more than 60 kg. Efficacy in promoting abstinence has not been demonstrated in patients who have not undergone detoxification or achieved alcohol abstinence. Therefore, acamprosate is indicated only in patients who are abstinent at the time of treatment initiation.

It does not undergo hepatic metabolism but is excreted as unchanged drug via the kidneys. The terminal half-life is approximately 20–33 hours. The pharmacokinetic profile of acamprosate is not altered in patients with hepatic disease or in alcohol-dependent patients. However, it can be altered by renal impairment. Peak plasma concentrations were 2 and 4-fold greater in patients with moderate and severe renal impairment, respectively, compared to healthy volunteers. The most common side effects of acamprosate are headache, diarrhea, flatulence and nausea.

### Other Pharmacotherapeutic drugs

In addition to the FDA approved drugs described above, there are several other drugs that show promise in treating alcohol addiction. Topiramate is one of such drugs, which is primarily used as an anticonvulsant. Chemically, it is a fructopyranosesulfamate. Although topiramate has not yet received FDA approval for treating alcohol addiction, it is sometimes used off-label for this purpose. It has been shown that topiramate significantly improves multiple drinking outcomes, as compared to a placebo^[16]^. The precise mechanism of action of topiramate is not known. It is thought to work by increasing inhibitory GABA neurotransmission at GABA_A_ receptors and reducing stimulatory glutamate neurotransmission at some types of glutamate receptors. Whereas kainate/AMPA (alpha-amino-3-hydroxy-5-methylisoxazole-4-propionic acid) subtype of excitatory glutamate receptor is antagonized by topiramate, no effect is observed on the activity of N-methyl-D-aspartate (NMDA) at the NMDA receptor.

Topiramate is administered orally. It is not metabolized to a great extent. About 70 – 80% of an administered dose is eliminated unchanged in the urine. It undergoes hydroxylation, hydrolysis, and glucuronidation reactions to generate about six metabolites, none of which constitutes more than 5% of an administered dose. Patients with hepatic impairments show altered pharmacokinetics of topiramate. There are significant side effect related to topiramate use including nervous system related paresthesia, cognitive dulling and fetal toxicity.

Baclofen is a derivative of γ-amino butyric acid (GABA). It is a GABA_B_ agonist that can inhibit the dopaminergic response to ethanol. Studies indicated that baclofen has positive benefit in abstinence, craving and daily alcohol intake^[17–19]^.

Kudzu is a natural medication mentioned by the National Institute on Alcohol Abuse and Alcoholism (NIAAA) for treating alcohol dependence. It is prepared from the Chinese herb *Puerarialobata*. Although there are contradictory results on its effectiveness in treating AUD and the mechanism of action is not well established, it is believed that the several isoflavone compounds present in Kudzu could inhibit aldehyde dehydrogenase^[20]^. It has been recently shown that dihydromyricetin, a flavonoid purified from Hovenia, has unique effects on GABA_A_ receptors and blocks ethanol intoxication and withdrawal in alcoholic animal models^[21]^.

## Summary & Conclusion

Despite the number of deaths and huge economic burden caused to the society, the number of drugs available to treat AUD is very limited and their mechanisms of action are not clearly understood. Moreover, the current drugs are only effective modestly and patient compliance is a serious issue because of several adverse side effects. Therefore, there is a need for discovery/development of new drugs. However, there are several challenges involved in the drug discovery/developments of AUD. Unlike other drugs of abuse where the drug target is more or less well defined, alcohol has multiple targets and is a low affinity ligand to most of these protein targets. Even, alcohol is known to target lipid membrane. Therefore, rational drug design for alcohol dependence has been proved to be extremely difficult. Another issue is the complex etiology of the disease and the heterogeneity of the AUD patients. The future of the drug development efforts will remain in the identification new alcohol targets^[22,23]^ and elucidating the molecular mechanism of its action is needed for effective intervention. In addition, current treatment could be improved by testing the existing medications for comorbid conditions using the patient’s genetic background information.

## Figures and Tables

**Figure 1:**
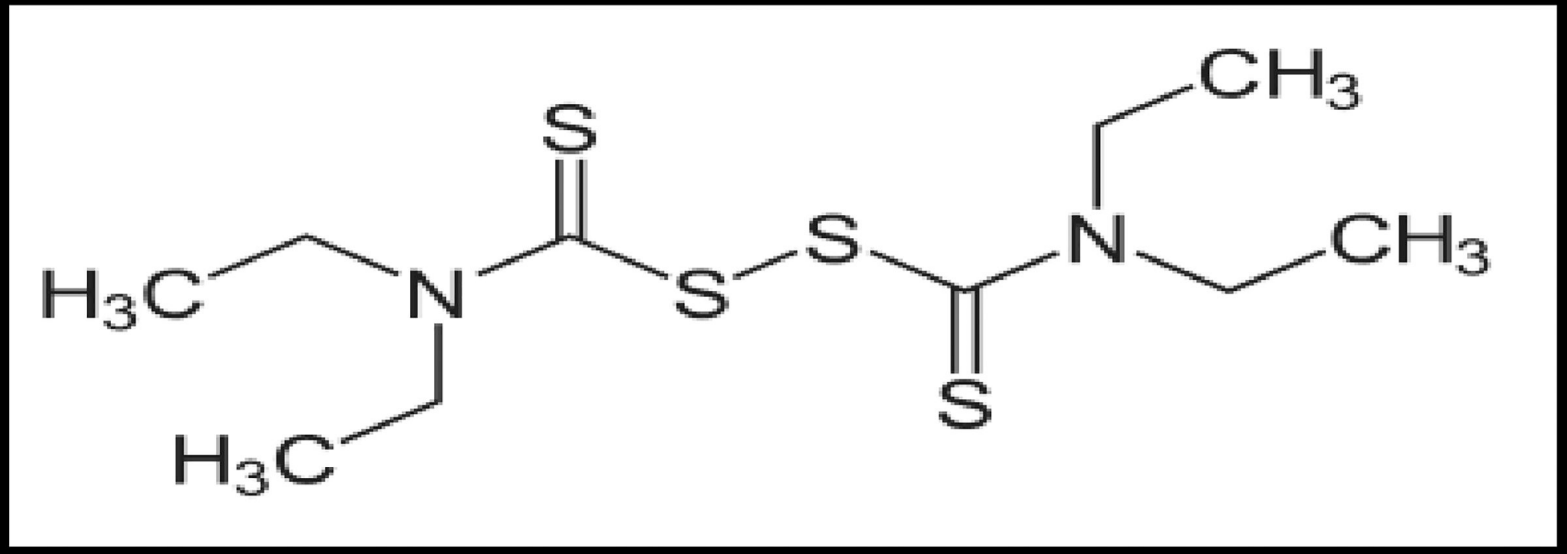
Disulfiram

**Figure 2:**
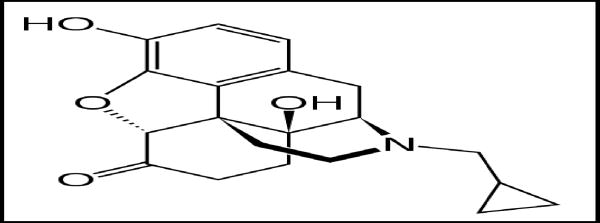
Naltrexone

**Figure 3:**
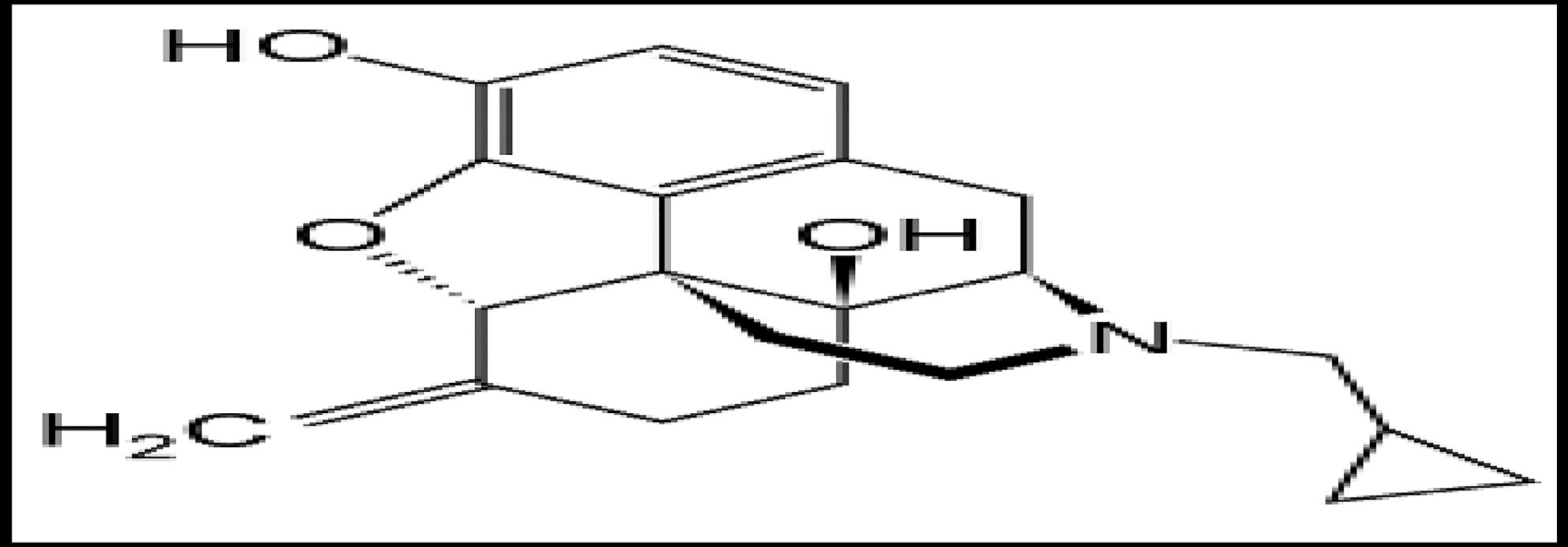
Nalmefene

**Figure 4:**
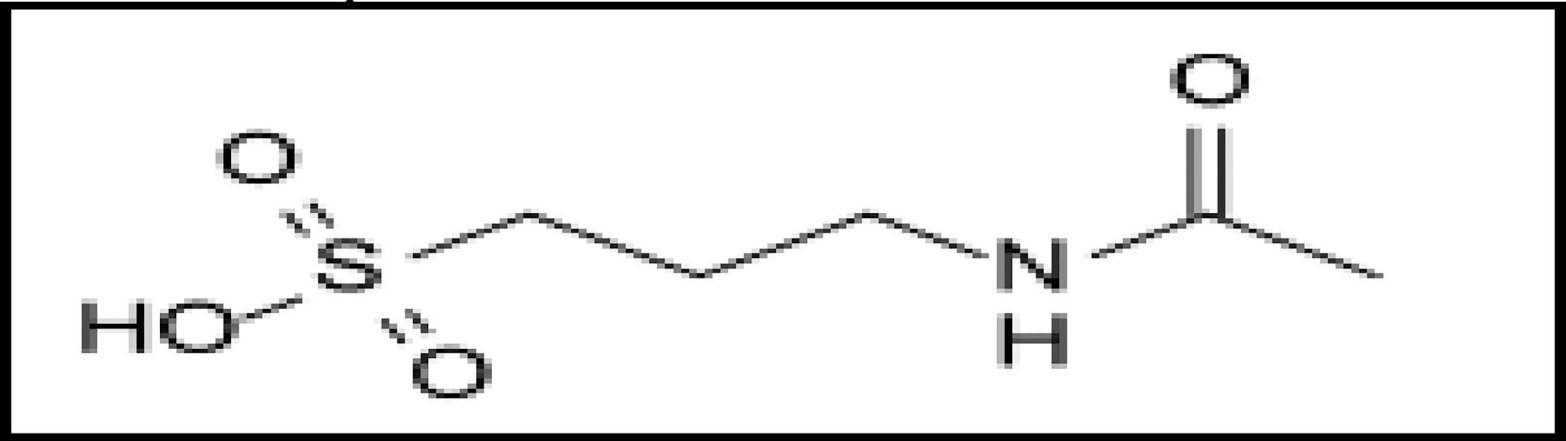
Acamprosate

**Figure 5:**
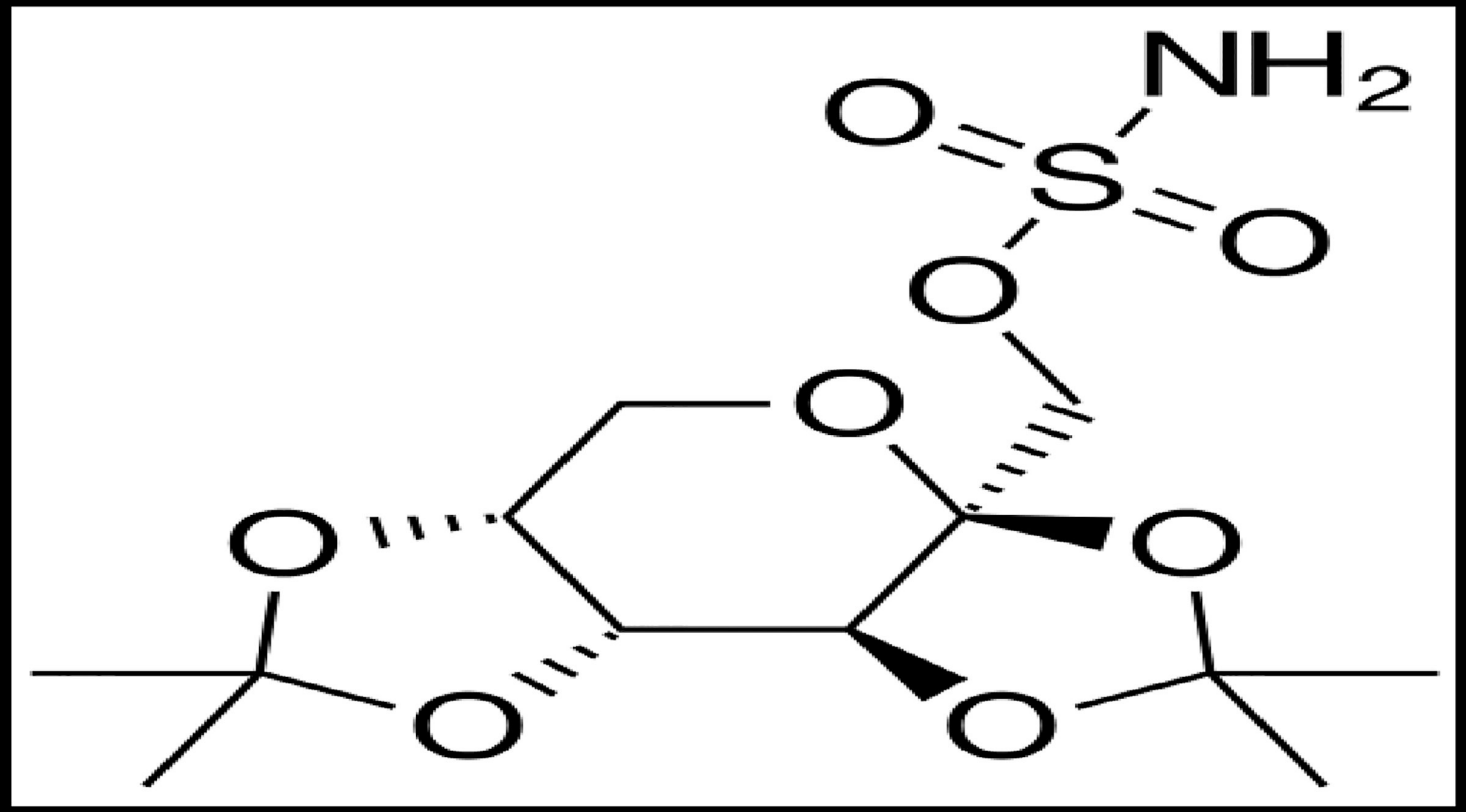
Topiramate

**Figure 6:**
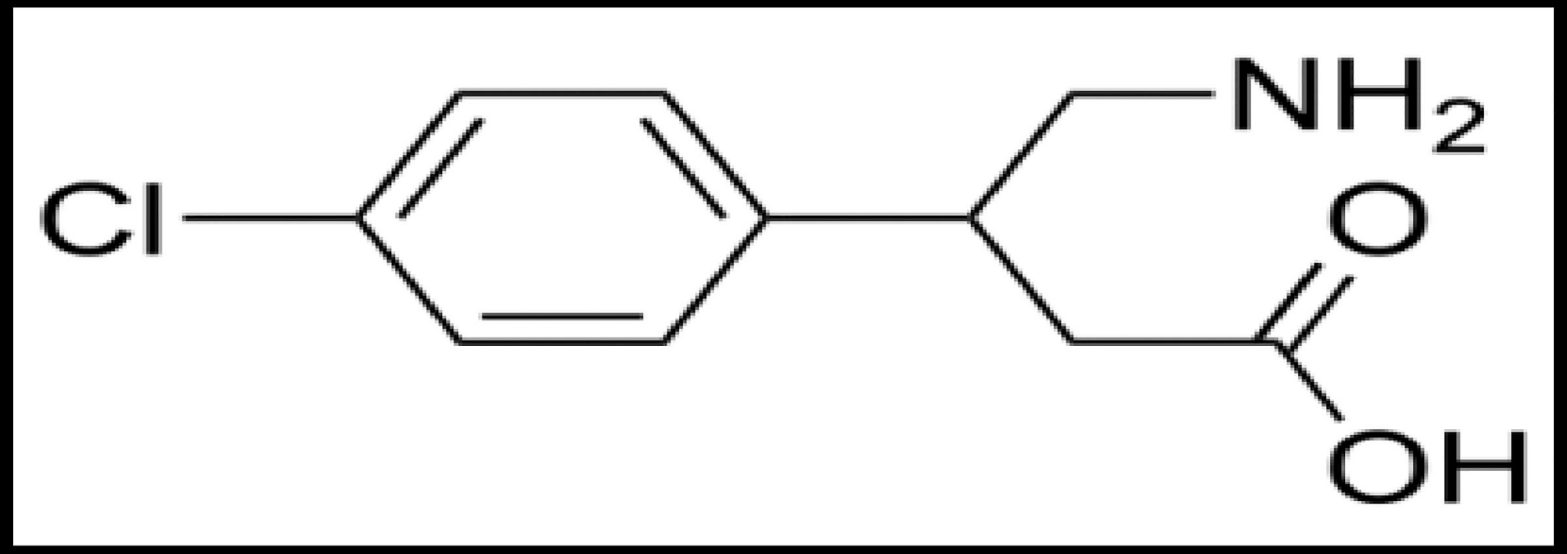
Baclofen

**Figure 7:**
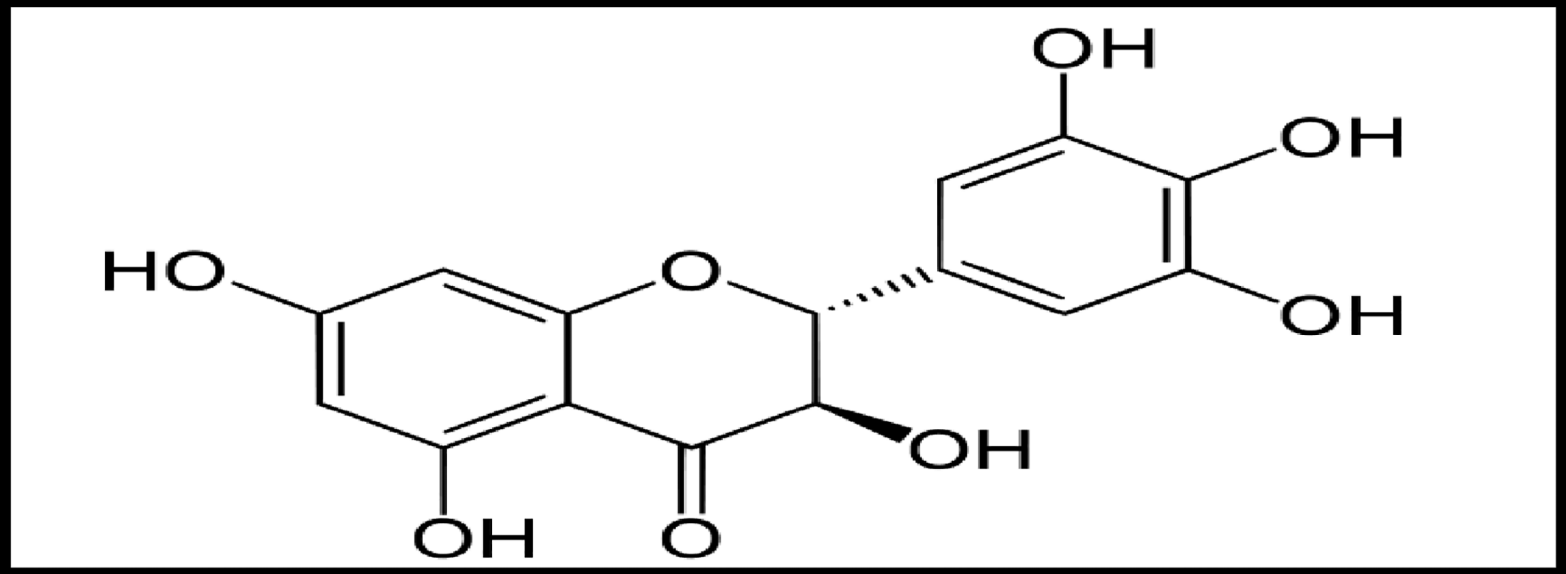
Dihydromyricetin
